# The Role of Village Doctors in Residents’ Uptake of Eye Screening: Evidence from Ageing Residents in Rural China

**DOI:** 10.3390/healthcare10071197

**Published:** 2022-06-26

**Authors:** Juerong Huang, Kang Du, Hongyu Guan, Yuxiu Ding, Yunyun Zhang, Decai Wang, Huan Wang

**Affiliations:** 1 College of Economics and Management, China Agricultural University, Beijing 100083, China; jrhuang0@163.com; 2 College of Economics, Xi’an University of Finance and Economics, Xi’an 710100, China; dukangceee@163.com; 3 Center for Experimental Economics in Education, Shaanxi Normal University, Xi’an 710119, China; dingyuxiuceee@163.com (Y.D.); zhangyunyunceee@163.com (Y.Z.); 4 Zhongshan Ophthalmic Center, State Key Laboratory of Ophthalmology, Sun Yat-sen University, Guangzhou 510060, China; 13622213157@163.com; 5 Center on China’s Economy and Institution, Stanford University, Stanford, CA 94305, USA; huanw@stanford.edu

**Keywords:** village doctor, eye care, eye screening, ageing population, cataracts, rural China

## Abstract

The lack of formal eye screening is the main reason for insufficient eye care utilization in rural China. Cataract, in particular, is increasingly prevalent with the aging population, but the treatment rate is relatively low. Village doctors are the most accessible health care resource for rural residents, receiving few empirical investigations into their role in eye care. This study aims to assess the role of village doctors in residents’ uptake of eye screening (vision and cataract screening), the first step of cataract treatment. Data come from a community-based, cross-sectional survey conducted in 35 villages of a county of the Gansu Province, Northwestern China, in 2020. Among 1010 residents aged ≥ 50 and 35 village doctors, the multivariate logistic regression shows that village doctors’ age, time spent on public health service, and service population were positively associated with residents’ uptake of vision and cataract screening. Village doctors were capable of playing an active role in primary eye health services due to their richer knowledge about cataracts than residents (accuracy rate 86.75% vs. 63.50%, *p* < 0.001), but less than half of them were willing to undertake eye screening. This study highlights the positive role of village doctors in aging residents’ eye screening and the potential role in improving the uptake of eye screening by offering health education.

## 1. Introduction

Eye care is a low-cost healthcare investment that boosts productivity and lowers social costs [1]. Access to eye care is critical for managing the progression of eye disease and treatment [2,3]. However, inadequate eye care utilization is still widespread in rural China. Cataract, in particular, is increasingly prevalent with the ageing population, but the cataract surgical coverage is relatively low [4,5]. A meta-analysis reports that China’s overall cataract surgery coverage rate is 9.19% [6], lower than the World Health Organization’s minimum criteria for eliminating cataract-related blindness [1]. Although Chinese rural residents have access to a certain percentage of reimbursement for cataract surgery through the New Rural Cooperative Medical Care, the cataract surgical rate is much lower among rural patients than urban patients [7]. People with cataracts left untreated will experience severe vision impairment, leading to lower quality of life, such as limited daily routines, reduced productive activities and lower income, even increasing the burden on other family members and society [8,9,10]. In the coming decades, if the projected increase in older people is not met with eye care services, there will be a substantial increase in the number of people with vision impairment and blindness in rural China.

The lack of formal eye screening is the main reason for inadequate eye care utilization in rural areas. Screening is the first step of eye disease treatment and may be the only timely way to identify vision disorders in rural areas [11,12]. By implementing eye screening programs, studies have shown that eye screening is a cost-effective investment [13,14]. However, eye screening only occurs informally in rural China, especially for middle-aged and older people. Besides that, rural residents also face greater eye screening barriers due to poor eye health literacy and a scarcity of trained optometrists [15,16]. Practical, effective eye screening models are required in rural China.

Village doctors (VDs) are the most accessible healthcare resource for rural residents [17]. The health care delivery system in rural China operates as a three-tiered structure: county hospitals, township health centers, and village clinics [18]. VDs deliver essential public health services in rural areas, such as disease prevention, maternal and child healthcare, health promotion, blood pressure control, and primary treatment of common and minor diseases [19,20,21]. As existing VDs retire and many younger clinicians go to cities, the number of VDs has decreased from over 1 million in 2010 to less than 0.8 million by 2020 [22,23]. Among VDs who remain, recent studies have shown concerns about the quality of services they provide [24,25]. Despite these recent deteriorations, VDs still play an irreplaceable role in rural primary health care, particularly for the rural poor and the elderly [17,26]. However, there have been few empirical investigations into the role of VDs in eye care. As the gatekeepers for rural residents’ health, whether VDs can help improve people’s eye screening and the accessibility of eye care in rural areas is the main content of this paper. The specific objectives of this paper are as follows: first, to explore whether VDs are associated with rural residents’ uptake of eye screening (vision and cataracts screening); second, to study VDs and residents’ knowledge about cataracts; and third, to investigate VDs’ willingness and attitudes towards providing eye screening services.

## 2. Materials and Methods

### 2.1. Setting

This community-based, cross-sectional study was conducted among adults aged 50 and older in rural areas of Q County in the eastern part of Gansu Province, Northwestern China. Gansu province is relatively underdeveloped compared to other regions of China. Official statistics indicate that in 2020, rural residents’ per capital income in Gansu was 39.6% lower than at the national level [27]. The rural residents’ per capital income in Q County was 2.5% lower than in Gansu province. Therefore, our sample area represents the situation in poorer rural areas.

### 2.2. Sampling

The study sample was collected using a random sampling procedure at the village level. First, a list of the population in each village was obtained through the local county people’s hospital, involving 153 villages. Ten villages were excluded due to the total population of the village being less than 800. Then, we randomly sampled a quarter of the villages and included 35 villages in this study. Finally, all residents aged ≥50 and VDs enrolled in villages participated in the survey. One village doctor in each village participated in our survey. If multiple VDs were in a village, the principal one involved.

Eligibility criteria included being a registered resident of Q, aged 50 and older, able to give oral informed consent, and verbally answering questions on the researcher-administered questionnaire. A total of 1049 participants were eligible for the study; 39 of them failed to complete the questionnaire. The response rate was 96.3%. Therefore, 1010 rural residents and 35 VDs were included in the study.

### 2.3. Data Collection and Measurement

The data collection included two parts: a questionnaire survey and eye screening. First, the questionnaire survey collected information on residents and VDs in the form of face-to-face interviews. The outcome variables of interest, rural residents’ uptakes of eye screening (vision and cataract screening) were measured by one’s self-reported answers. Specifically, uptake of vision and cataract screening was measured by asking whether they had undergone vision screening and cataract screening before the time of the survey, respectively. We construct two dummy variables to measure whether a resident had undertaken vision or cataract screening (=1 if yes; =0 if no).

The survey also collected residents’ demographic and socioeconomic information (Table 1), including age, gender, education status, marital status, whether the interviewee lives with at least one child, and annual household income. Residents’ knowledge about cataracts was assessed using eight questions (detailed information shown in Table 2). Each item was equally weighted. The eight dichotomous variables (knowledge about cataracts) were given a score of 0 (wrong answer) or 1 (right answer).

Another important piece of information provided in this survey is VDs’ characteristics (Table 1), which facilitates us to examine the role of VDs in residents’ uptake of eye screening. First, information on VDs’ characteristics was obtained, including age, gender, education status, working experience, whether they lived in the village they worked, and work time spent on the public health services, the village clinic’s service population, and service radius. Secondly, VDs’ knowledge about cataracts was collected and measured in the same way as the residents’ questionnaire (Table 2). Finally, we also asked about the VDs’ willingness and attitude toward providing services for vision and cataract screening. 

Second, this study also carried out the eye screening (vision and cataract screening) by a team of three members, including an ophthalmologist and a nurse from the local public hospital, and an assistant. VDs informed residents (aged ≥ 50) to participate in the eye screening for free three days in advance. Residents come to the village clinic for screening between 9 a.m. to 5 p.m. on the appointed day. The participants’ uncorrected VA was measured by Early Treatment Diabetic Retinopathy Study (ETDRS) Tumbling E charts (Precision Vision, La Salle, IL, USA), a worldwide standard instrument [28]. VA was assessed separately for each eye without refractive correction at 4 m using the ETDRS charts. The ophthalmologists also examined residents with a slit lamp to determine if they have any opacity of the crystalline lens in either eye, the criteria for cataracts [4,29]. Moreover, we generate a dummy variable to measure whether VA in either eye is ≤0.3 (0 = no, 1 = yes), which is the recommended criteria for cataract surgery [30].

### 2.4. Ethical Approval

Ethical approval for this study was granted by the Stanford University Institutional Review Board (Protocol ID 64729). We adhered to the Declaration of Helsinki throughout the survey in terms of both maintaining privacy and ensuring confidentiality. Oral informed consent was obtained from all participants. Participants whose VA ≤ 0.3 were referred to local county hospitals for treatment.

### 2.5. Statistical Analysis

Residents’ and VDs’ characteristics, knowledge about cataracts, and VDs’ willingness and attitude toward providing eye services were described using proportions. The uptake rates of vision and cataract screening were tabulated by age. Multivariate logistic analyses were used to analyze the determinants of eye screening. Odds ratios (OR), 95% confidence intervals (CI), and *p*-value are presented in Table 3. All analyses were conducted by using Stata15.1 (StataCorp, College Station, TX, USA).

## 3. Results

### 3.1. Characteristics of Residents and Village Doctors

Table 1 presents summary statistics for the rural residents and VDs. Among the 1010 residents (60% female; mean age 62.75 ± 1.0 years), 44% did not receive formal education; 33% and 23% completed primary and secondary education. 74% of them had a spouse at the time of the survey. Less than half (45%) of those residents lived with at least one child. The mean of the log annual household income (RMB, yuan) was 8.95 ± 1.14. 34% of sample residents’ visual acuity was less than 0.3 in either eye, and 44.65% were diagnosed with cataracts.

Among 35 village doctors (77% male; mean age 46.37 ± 1.0 years), 82% and 17% of them had completed high school and college, respectively. 66% of them lived in the village they worked. 63% of VDs worked for more than ten years. Most (77%) of them spent more than half of their work time on public health service last year. 65% of VDs provided health services for less than 2000 people; 80% of VDs’ service radius was within 10 km.

### 3.2. Uptake Rate of Residents’ Vision Screening and Cataract Screening

**Figure 1:**
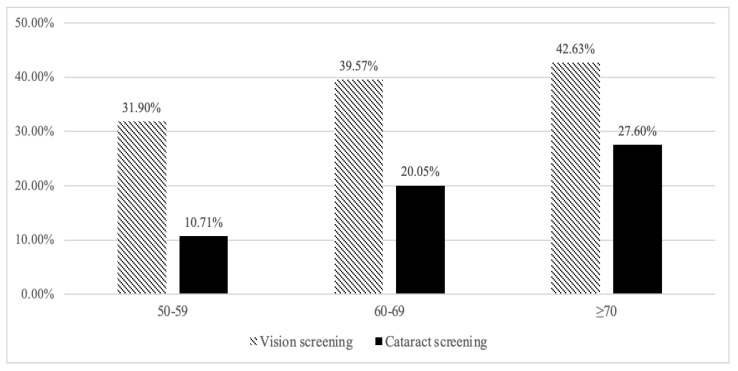
Age-specific Uptake Rate of services for Vision Screening and Cataract Screening.

The uptake rate of vision and cataract screening was 37.00% and 17.80%, respectively. The age-specific uptake rates are shown in Figure 1. Specifically, among residents aged 50–59, 31.90% of them had taken vision screening, but only 10.71% had received cataract screening. The vision and cataract screening uptake among residents aged 60–69 was 39.57% and 20.05%, respectively. Among residents aged 70 and older, 42.63% of them had done vision screening, but only less than one-third (27.60%) had taken cataract screening. In short, the uptake rate of services for vision and cataract screening increased with age but remains fairly low.

### 3.3. Determinants of Residents’ Uptake of Vision Screening

Table 3 presents the determinants associated with residents’ uptake of services for eye screening: vision screening (column 1) and cataract screening (column 2). Multiple logistic regression analysis revealed that the following VDs’ characteristics were positively associated with a higher vision screening uptake of residents: older age (*p* = 0.064), living in the same village they worked (*p* = 0.086), more than half of work time spent on public health service (*p* = 0.009), smaller service population (*p* = 0.003). 

In addition, residents with older age (*p* < 0.1), female (*p* = 0.079), secondary education and above (*p* = 0.001) and visual acuity ≤ 0.3 (*p* = 0.036) were more likely to undergo a vision screening.

### 3.4. Determinants of Residents’ Uptake of Cataract Screening

Turning to cataract screening (column 2, Table 3), VDs’ characteristics were also positively associated with residents’ uptake of cataract screening: older age (*p* = 0.001), male ((*p* < 0.001), work experience over ten years (*p* = 0.059), more than half of work time spent on public health service (*p* = 0.015), and smaller service population (*p* = 0.006). Moreover, residents with older age (*p* < 0.01) and VA ≤ 0.3 in either eye (*p* < 0.001) were more likely to undergo a cataract screening. 

### 3.5. Residents’ and Village Doctors’ Knowledge about Cataracts

Since unawareness and misunderstanding may hinder residents from seeking eye care services [31], we further investigated the knowledge of residents and VDs on cataracts. Table 2 reveals the knowledge gap on cataracts between residents (mean score 5.08 ± 1.91) and VDs (mean score 6.94 ± 1.14). Chi-square tests suggest this gap significantly exists (*p <* 0.001).

Residents had basic knowledge about cataracts, but an inadequate understanding of how to treat cataracts. Specifically, more than two-thirds of residents (69.5%) believed that cataracts are a common disease; 79.9% believed regular cataract screening should be undertaken. Most of them had a correct understanding of cataract surgery, i.e., surgery can cure cataracts (75.0%), surgery is safe (71.9%), and surgery can improve vision (71%). However, only 48% knew that medicine cannot treat cataracts; 39% knew that cataract surgery should not be delayed until blind. Although having access to the reimbursement for cataract surgery through the New Rural Cooperative, only 54% of residents knew this information. 

VDs’ knowledge of cataracts was much better than residents. More than 90% of VDs had a correct understanding of cataracts. Only one question (i.e., cataract surgery should not be delayed until blind) was answered correctly by 88% of VDs. 

### 3.6. Village Doctors’ Willingness and Attitude towards Providing Eye Screening Services

**Figure 2:**
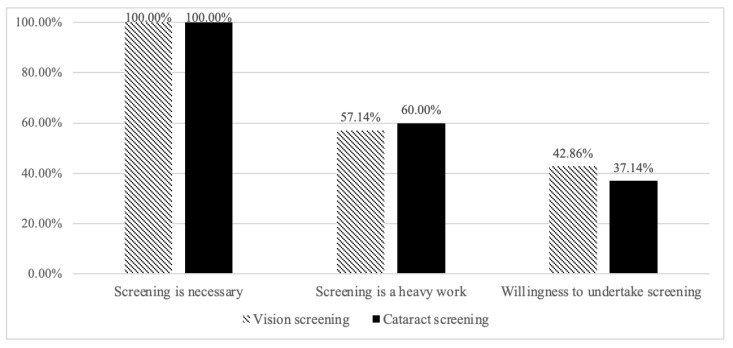
Village doctors’ willingness and attitude towards providing services for vision or cataract screening (N = 35), Percentage (%).

We further investigated VDs’ willingness and attitudes towards providing eye care services. Figure 2 shows that all VDs thought that vision and cataract screening were necessary. However, most VDs thought that vision (58%) and cataract screening (60%) were heavy work. When asked about their willingness to screen, only 46% and 37% of VDs were willing to undertake vision and cataract screening. 

## 4. Discussion

This study presents three main findings by investigating the role of VDs. First, given the low uptake of services for eye screening, VDs are significantly associated with residents’ uptake of vision and cataract screening. Second, compared with residents, VDs have relatively comprehensive and correct knowledge about cataracts. Third, though all agreed with the necessity to provide eye screening, less than half of VDs were willing to do so. We discuss this in detail as follows.

Screening is well acknowledged as the prerequisite for detecting potential eye disease and seeking treatment [13]. The World Health Organization recommends integrating screening into existing health delivery systems [1]. However, in China, vision screening and cataract screening are still not the basic public services provided by the government health sector. In our study, less than 40% and 20% of the residents had ever received a vision and cataract screening, which is not a particular case in other rural areas of China [32,33].

As the gatekeepers for rural residents’ health, VDs are associated with residents’ uptake of eye screening services. In particular, rural residents were more likely to undertake vision and cataract screening in areas where VDs were older, spent more time on public health services, and serviced a smaller population. In addition, VDs’ other characteristics are also positively associated with residents’ uptake of cataract screening: male, more experienced, and smaller service radius.

VDs are capable of playing an active role in primary eye care due to their richer knowledge about cataracts than residents. For example, our results suggest that all VDs knew that the New Cooperative Medical System covers cataract surgery, but only 54% of residents knew this. Studies showed that enriching eye-related knowledge could improve eye screening [34,35]. In rural areas, VDs are trusted by villagers and are always the first choice for seeking health services [36,37]. They can communicate promptly with residents because most (2/3) of them live in the same village, which means the knowledge gap may be narrowed by information transfer from VDs to rural residents. Studies in other countries have also found that primary care providers (similar to village doctors) can play a role in improving community residents’ eye health knowledge [38,39]. Many efforts aiming at enhancing the uptake of eye care services in rural regions involve VDs, particularly in improving residents‘ eye health awareness and referring patients with eye disorders [40].

The willingness of village doctors to provide services of eye (vision and cataract) screening is relatively low. Figure 2 shows that all VDs agreed with the necessity of providing vision and cataract screening services, but less than half of them were willing to undertake it. Based on our interviews with VDs, the unwillingness was mainly due to two reasons: first, VDs did not know how to perform eye screening, including vision and cataract screening; second, VDs thought they already had a heavy workload and hoped to get some extra subsidies. Evidence suggests that increasing the public health responsibilities of VDs requires a considerable time investment (e.g., the management of non-communicable illnesses) [41]. Despite the fact that VDs cannot directly provide eye screening services, our research found that due to their proximity to residents and better eye care knowledge, VDs may be able to improve residents’ utilization of eye screening by providing eye education and encouraging residents to go for eye screening.

This study has potential limitations owing to sample selection and data collection. First, the key explained variable in our study is measured by asking whether residents participated in vision or cataract screening. In particular, the actual time and frequency of screening are not available in the data. Future studies may benefit from constructing more specific and detailed measures that capture residents’ eye screening more accurately. Second, the entire sample came from rural areas in the North-western province (Gansu) of China, which provides a reference for underdeveloped regions. In addition, the small sample size of village doctors in this study may limit the representativeness of our results. Multi-centrality research encompassing diverse regions in the future is likely to be fruitful in deepening our understanding of the role of village doctors in residents’ eye screening. Despite these limitations, we believe that our study provides new and helpful information to inform policy.

Given the stratified medical system in China, investing in VDs might be cost-effective due to lower communication and labor costs. For example, compared with higher-level institutions (e.g., county hospitals), VDs are familiar with villagers and more effortless to convey information, reminder, and education. As most of them live closely with residents, so they have lower labor costs and living costs [42]. Therefore, our findings have at least three important policy implications. First, eye screening should be included in the basic public health service system for rural populations. For example, the township health center may provide regular vision screening to residents over 50 and refer those with VA ≤ 0.3 to the county hospital for further cataract screening. Second, policymakers should emphasize the importance of VDs in primary eye care, encourage VDs to participate in eye health education, and motivate residents to check their eyes. Third, given their potential capacity to deliver eye health care, the quantity and quality of VDs need to be improved.

## 5. Conclusions

As the most accessible healthcare resource for rural residents, residents’ uptake of eye-screening services is highly correlated with VDs. Given that VDs have richer knowledge about cataracts than residents, they are capable of playing a more active role in primary eye care. But VDs have a relatively low willingness to directly provide eye screening services. The role of VDs, as an efficient and low-cost human resource in eye care, should be emphasized, especially in rural China and other low and middle-income countries. This study contributes to the current literature that discusses the role of VDs in the Primary Health Service System.

## Figures and Tables

**Table 1:** Demographic characteristics of participants.

Characteristics	N	Percent (%)
**Characteristics of Residents (*n* = 1010)**		
Age (years)		
50–59	420	41.58
60–69	369	36.53
≥70	221	21.88
Gender		
Female	604	59.80
Male	406	40.20
Education status		
No formal education	444	43.96
Primary education	332	32.87
Secondary education and above	234	23.17
Marital status		
Do not have a spouse	262	25.94
Have a spouse	748	74.06
Live with at least one child		
No	556	55.05
Yes	454	44.95
Annual household income (log), Mean (SD)	1010	8.95 (1.14)
Visual acuity ≤ 0.3 in either eye		
No	663	65.64
Yes	347	34.36
**Characteristics of VDs (*n* = 35)**		
Age (years) of the VD		
<45	13	37.14
≥45	22	62.86
Gender of the VD		
Female	8	22.86
Male	27	77.14
Education status of the VD		
High school	29	82.86
College	6	17.14
Whether the VD lives in the village		
No	12	34.29
Yes	23	65.71
Work experience (years) of the VD		
<10	13	37.14
≥10	22	62.86
More than half of work time spent on public health services last year		
No	8	22.86
Yes	27	77.14
Service population		
<2000	23	65.71
≥2000	12	34.29
Service radius (KM)		
<10	28	80.00
≥10	7	20.00

**Table 2:** Knowledge about cataract among residents (*n* = 1010) and VDs (*n* = 35).

Questions	Scoring Scheme	Correctly Answered by the Residents	Correctly Answered by the VD
		N (%)	N (%)
1. Should the elderly do regular cataract screening?	Yes = 1 mark	807 (79.90)	32 (91.43)
2. Can cataracts be cured by surgery?	Yes = 1 mark	757 (74.95)	33 (94.29)
3. Whether cataract surgery is safe?	Yes = 1 mark	726 (71.88)	34 (97.14)
4. Whether cataract is a common disease for elders?	Yes = 1 mark	702 (69.50)	32 (91.43)
5. Will cataract surgery improve eyesight?	Yes = 1 mark	721 (71.39)	34 (97.14)
6. Can cataracts be cured with medications?	No = 1 mark	485 (48.02)	32 (91.43)
7. Whether cataract surgery is covered by the New Cooperative Medical System?	Yes = 1 mark	536 (54.06)	35 (100.00)
8. Should a patient delay cataract surgery before being blind?	No = 1 mark	392 (38.81)	31 (88.57)
Mean (SD)	-	5.08 (1.91)	6.94 (1.14)

**Table 3:** Determinants of residents’ uptakes of services for eye screening: vision screening and cataract screening.

Variables	Vision Screening (1)		Cataract Screening (2)	
	OR (95% CI)	*p* Value	OR (95% CI)	*p* Value
**Characteristics of residents (*n* = 1010)**				
Age				
50–59 (ref)				
60–69	1.52 (1.00–2.33)	0.051	1.84 (1.26–2.69)	0.002
≥70	1.61 (0.97–2.69)	0.063	2.19 (1.24–3.87)	0.007
Gender				
Female (ref)				
Male	0.74 (0.53–1.04)	0.079	0.83 (0.59–1.16)	0.271
Education status				
No formal education (ref)				
Primary education	1.46 (0.91–2.33)	0.116	0.81 (0.43–1.53)	0.510
Secondary education and above	2.08 (1.35–3.19)	0.001	1.56 (0.85–2.86)	0.150
Marital status				
Do not have a spouse (ref)				
Have a spouse	0.97 (0.63–1.49)	0.892	0.80 (0.53–1.18)	0.248
Live with a least one child				
No (ref)				
Yes	0.82 (0.60–1.11)	0.190	0.87 (0.58–1.30)	0.491
Annual household income (log)	0.95 (0.84–1.07)	0.379	0.92 (0.82–1.04)	0.177
Visual acuity ≤ 0.3 in either eye				
No (ref)				
Yes	1.39 (1.02–1.90)	0.036	2.08 (1.42–3.05)	0.000
**Characteristics of VDs (*n* = 35)**				
Age (years) of the VD				
<45 (ref)				
≥45	1.45 (0.98–2.15)	0.064	1.75 (1.24–2.47)	0.001
Gender of the VD				
Female (ref)				
Male	1.20 (0.87–1.66)	0.265	3.09 (2.15–4.43)	0.000
Education status of the VD				
High school (ref)				
College	1.13 (0.56–2.31)	0.728	1.20 (0.71–2.04)	0.495
Whether the VD lives in the village				
No (ref)				
Yes	1.59 (0.94–2.70)	0.086	1.17 (0.77–1.76)	0.461
Work experience (years) of the VD				
<10 (ref)				
≥10	0.72 (0.41–1.27)	0.259	0.65 (0.41–1.02)	0.059
More than half of work time spent on public health services last year				
No (ref)				
Yes	1.94 (1.18–3.21)	0.009	1.64 (1.10–2.44)	0.015
Service population				
<2000 (ref)				
≥2000	0.61 (0.44–0.85)	0.003	0.62 (0.44–0.87)	0.006
Service radius (KM)				
<10 (ref)				
≥10	1.35 (0.86–2.12)	0.196	0.76 (0.54–1.06)	0.101

## Data Availability

The datasets used and analyzed during the current study are available from the corresponding author on reasonable request.
